# Effect of *Centella asiatica* Leaf Extract on the Dietary Supplementation in Transgenic *Drosophila* Model of Parkinson's Disease

**DOI:** 10.1155/2014/262058

**Published:** 2014-11-27

**Authors:** Yasir Hasan Siddique, Falaq Naz, Smita Jyoti, Ambreen Fatima, Saba Khanam, Rahul, Fahad Ali, Syed Faiz Mujtaba, Mohammad Faisal

**Affiliations:** ^1^Drosophila Transgenic Laboratory, Section of Genetics, Department of Zoology, Faculty of Life Sciences, Aligarh Muslim University, Aligarh, Uttar Pradesh 202002, India; ^2^Photobiology Division, CSIR-Indian Institute of Toxicology Research, Lucknow, Uttar Pradesh 206002, India; ^3^Forest Entomology Division, Forest Research Institute, Dehradun 248006, India

## Abstract

The role of *Centella asiatica* L. leaf extract was studied on the transgenic *Drosophila* model flies expressing normal human alpha synuclein (h-*α*S) in the neurons. The leaf extract was prepared in acetone and was subjected to GC-MS analysis. *C. asiatica* extract at final concentration of 0.25, 0.50, and 1.0 *μ*L/mL was mixed with the diet and the flies were allowed feeding on it for 24 days. The effect of extract was studied on the climbing ability, activity pattern, lipid peroxidation, protein carbonyl content, glutathione content, and glutathione-S-transferase activity in the brains of transgenic *Drosophila*. The exposure of extract to PD model flies results in a significant delay in the loss of climbing ability and activity pattern and reduced the oxidative stress (*P* < 0.05) in the brains of PD flies as compared to untreated PD flies. The results suggest that *C. asiatica* leaf extract is potent in reducing the PD symptoms in transgenic *Drosophila* model of Parkinson's disease.

## 1. Introduction

*Centella asiatica* commonly known as Indian Penny Wort is found in the swampy areas of India. It is use in traditional Chinese and Ayurvedic medicine [1]. It can be consumed either fresh or may be processed into tea/juice for various ailments including wound healing, bronchitis, asthma, gastric, kidney troubles, urethritis, liver disorders, antiallergic, anticancer, hypotensive, and other ailments [2]. Parkinson's disease (PD) is a chronic neurodegenerative disorder characterized by the progressive loss of the dopaminergic neurons in substantia nigra pars compacta of mid brain [3]. Locomotor dysfunction is one of the clinical symptoms of PD and has been linked to the death of dopaminergic neurons [4]. The accumulation of Lewy bodies, loss of dopaminergic neurons, and climbing ability has been reported in transgenic flies [5]. The over expression of wild type *α*-synuclein is toxic to dividing cells and overexpression of its mutant forms A53T or A30P exhibit even greater toxicity [6]. Studies have been carried out on the oxidative stress and mitochondrial dysfunction, leading to neuronal dysfunction and ultimately cell death [7]. The study of the pathogenesis of PD has been rapidly enhanced by the development of different models and these models have been proved to be very useful for understanding the mechanism of neurodegeneration and possible strategy for neuroprotection [7]. Plants having medicinal properties have gained importance because of their beneficial effect on human health care [8]. In the present study the effect of* Centella asiatica* leaf extract was studied on the PD model transgenic flies.

## 2. Materials and Methods

### 2.1. Preparation of Leaf Extract

The leaves of* C. asiatica* were collected from the nursery of Forest Research Institute (FRI), Dehradun (Accession number 94517). The extract was prepared according to the protocol described in our earlier published work [9].

### 2.2. Analysis of* C. asiatica* Extract through GC-MS

GC-MS analysis was performed using Trace GC ultragas chromatograph connected to a Quantum XLS mass spectrometer (Thermo Scientific, FL, USA). GC was equipped with TG-5MS capillary column (30 m × 0.25 mm i.d. × 0.25 *μ*m film thickness) consisting of a stationary phase 5% phenyl and 95% methyl polysiloxane. The injection was carried out in CT split less mode at an injector temperature of 260°C. Helium gas was used as a carrier gas with a flow rate of 1.1 mL/min. The oven temperature programming was as follows: the initial oven temperature was held at 70°C for 2.0 min and then increased to 210°C at a rate of 20°C/min and then increased to 290°C at a rate of 10°C/min held for 13.0 min. The ion source and transfer line temperature were 220°C and 290°C, respectively. Identification of the compounds was performed by comparing their mass spectra with the NIST library available in the instrument.

### 2.3. *Drosophila* Stocks

Transgenic fly lines that expresses wild-type human synuclein (h-*α*S) under UAS control in neurons “(w[∗]; P{w[+mC] = UAS-Hsap/SNCA.F}”5B and GAL4 “w[∗]; P{w[+mC] = GAL4-elavL}”3 were obtained from Bloomington* Drosophila* Stock Center (Indiana University, Bloomington, IN). When the males of UAS- (Upstream Activation Sequence-) Hsap/SNCA.F strains are crossed with the females of GAL4-elav.L (vice-versa), the progeny will express the human *α*S in the neurons [5].

### 2.4. *Drosophila* Culture and Crosses

The flies were cultured on standard* Drosophila* food containing agar, corn meal, sugar, and yeast at 25°C (24 ± 1) [10]. Crosses were set up as described in our earlier published work [11]. First, the climbing assay was performed for the PD flies and UAS-Hsap/SNCA.F (control). The other group of PD flies was allowed feeding on separately to different doses of* C. asiatica* extract mixed in the diet.* C. asiatica* extract was added in the diet at final concentration of 0.25, 0.50, and 1.0 *μ*L/mL. The PD flies were also exposed to 10^−3^ M of L-dopamine. The UAS-Hsap/SNC.F act as a control. The control flies were separately allowed feeding on the selected doses of* C. asiatica* extract.

### 2.5. *Drosophila* Climbing Assay

The climbing assay was performed as described by Pendleton et al. [12]. Ten flies were placed in an empty glass vial (10.5 cm × 2.5 cm). A horizontal line was drawn 8 cm above the bottom of the vial. After the flies had acclimated for 10 min at room temperature, both controls and treated groups were assayed at random, to a total of 10 trails for each. The mean values were calculated and then averaged, and a group mean and standard error were obtained. All behavioral studies were performed at 25°C under standard lightning conditions.

### 2.6. *Drosophila* Activity Pattern

From the 12th day the activity of flies (males) in all treated groups was analyzed by using* Drosophila* Activity Monitor (TriTek, USA). The activity was recorded every hour for a total of 384 h and the data was analyzed by Actogram J software. The results were presented as chi-square periodogram [13].

### 2.7. Lipid Peroxidation Assay

Lipid peroxidation assay in the brain homogenate was performed according to the procedure described by Siddique et al. [10, 11]. Reagent 1 (R1) was prepared by dissolving 0.064 g of 1-methyl-2-phenylindole into 30 mL of acetonitrile to which 10 mL of methanol was added to bring the volume to 40 mL. The preparation of 37% HCl served as the reagent R2. The brain of flies were isolated under stereozoom microscope in ice cold Tris HCl (20 mM) (10 brain/group; five replicates/group). Homogenate was prepared in Tris HCl and centrifuged at 3000 g for 20 min and subsequently the supernatant was collected. In a microcentrifuge tube 1300 *μ*L of R1 was taken. A volume of 1 *μ*L of supernatant was added along with 300 *μ*L of R2 vortexed and incubated at 45°C for 40 min. After incubation, the tubes were cooled in ice and centrifuged at 15,000 g for 10 min at 4°C. All samples were read at 586 nm.

### 2.8. Estimation of Protein Carbonyl Content

The protein carbonyl content was estimated according to the protocol described by Hawkins et al. [14]. The brain homogenate was diluted to a protein concentration of approx 1 mg/mL. About 250 *μ*L of each diluted homogenate was taken in Eppendorf centrifuge tubes separately. To it 250 *μ*L of 10 mM 2,4-dinitrophenyl hydrazine (dissolved in 2.5 M HCl) was added, vortexed, and kept in dark for 20 min. About 125 *μ*L of 50% (w/v) trichloroacetic acid (TCA) was added, mixed thoroughly, and incubated at −20°C for 15 min. The tubes were then centrifuged at 4°C for 10 min at 9000 rpm. The supernatant was discarded and the pellet obtained was washed twice by ice cold ethanol : ethyl acetate (1 : 1). Finally the pellets were redissolved in 1 mL of 6 M guanidine hydrochloride and the absorbance was read at 370 nm.

### 2.9. Estimation of Glutathione (GSH) Content

The glutathione (GSH) content was estimated colorimetrically using Ellman's reagent (DTNB) according to the procedure described by [15]. The supernatant was precipitated with 4% sulfosalicylic acid (4%) in the ratio of 1 : 1. The samples were kept at 4°C for 1 hr and then subjected to centrifugation at 5000 rpm for 10 min at 4°C. The assay mixture consisted of 550 *μ*L of 0.1 M phosphate buffer, 100 *μ*L of supernatant, and 100 *μ*L of DTNB. The OD was read at 412 nm and the results were expressed as *μ* moles of GSH/gram tissue.

### 2.10. Estimation of Glutathione-S-Transferase (GST) Activity

The glutathione-S-transferase activity was determined by the method of Habig et al. [16]. The reaction mixture consists of 500 *μ*L of 0.1 M phosphate buffer, 150 *μ*L of 10 mM CDNB, 200 *μ*L of 10 mM reduced glutathione, and 50 *μ*L of supernatant. The OD were taken at 340 nm and the enzyme activity was expressed as *μ* moles of CDNB conjugates/min/mg protein.

### 2.11. Statistical Analysis

The statistical analysis was done using Statistica Soft Inc. The mean values of various fly groups were statistically compared using an unpaired group of the Student *t*-test.

## 3. Results

**Figure 1:**
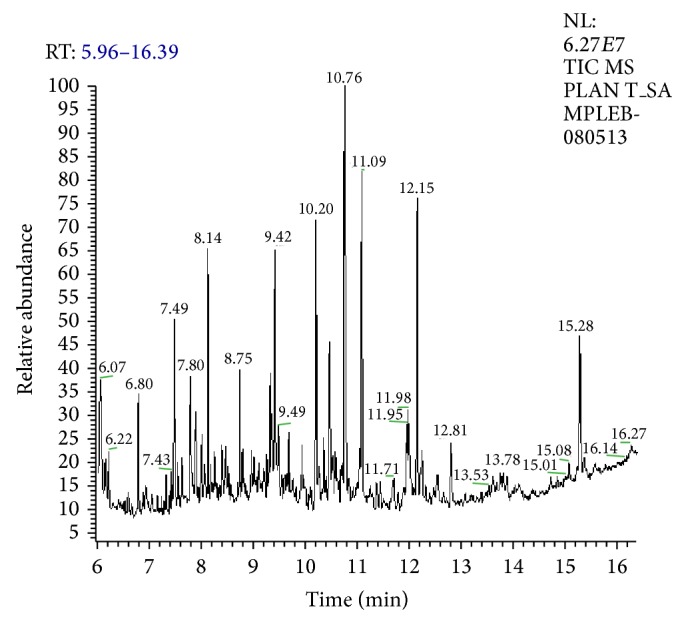
GC chromatogram of the leaf extract (acetone) of* Centella asiatica*.

**Figure 2:**
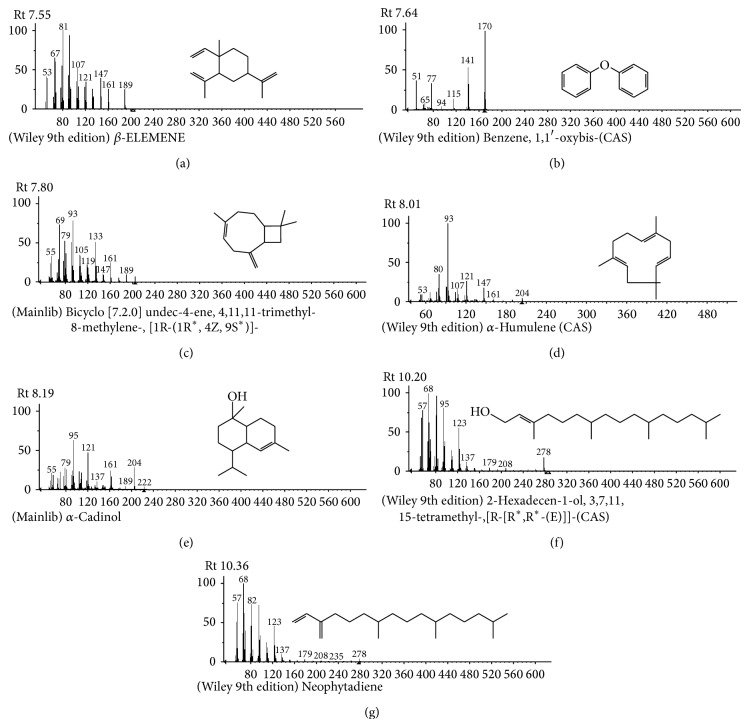
(a)–(g) Mass spectra of acetone extract with its products having the high concentration compared with NIST library.

**Figure 3:**
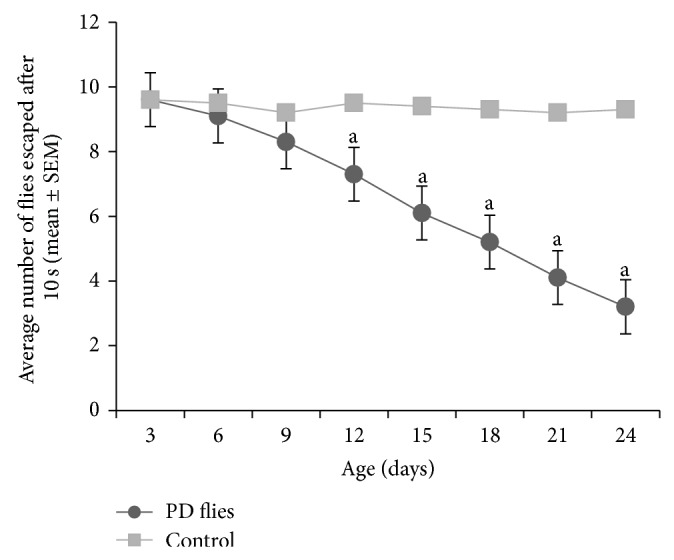
Climbing ability in Parkinson disease (PD) flies and control for a period of 24 days. The values are the mean of five assays (^a^significant with respect to control *P*-0.01). Control flies: UAS-alpha-syn; PD flies: elav-GAL4; UAS-alpha-syn.

**Figure 4:**
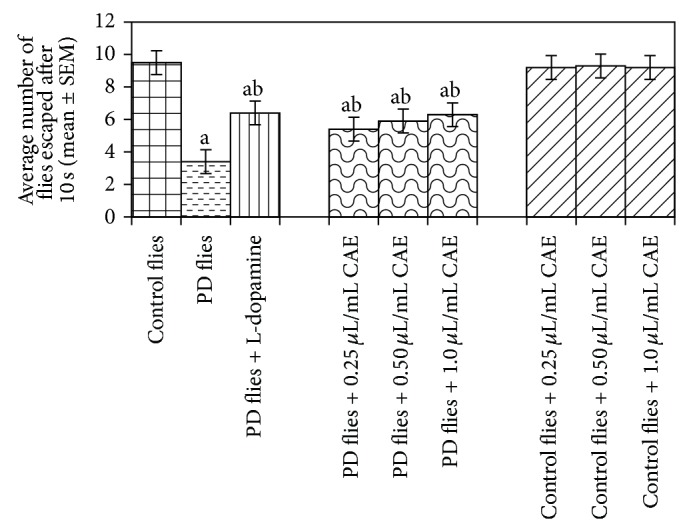
Effects of* Centella asiatica* extract (CAE) on the climbing ability. The flies were allowed feeding on the diet supplemented with* Centella asiatica* extract (CAE) for 24 days and then assayed for climbing ability. The values are the mean of five assays. (^a^significant with respect to control, *P*-0.01; ^b^significant with respect to PD model flies, *P*-0.05). L-Dopa = 10^−3^ M; control flies: UAS-alpha-syn; PD flies: elav-GAL4; UAS-alpha-syn.

**Figure 5:**
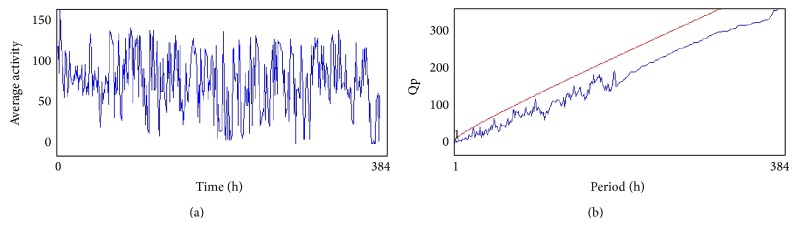
(a) and (b) show the average activity pattern and chi-square periodogram, respectively, for the control flies (*N* = 20).

**Figure 6:**
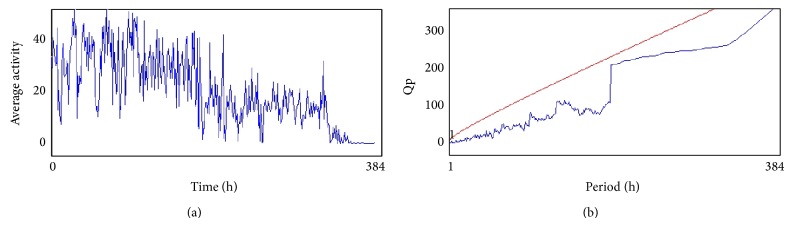
(a) and (b) show the average activity pattern and chi-square periodogram, respectively, for the PD flies (*N* = 20).

**Figure 7:**
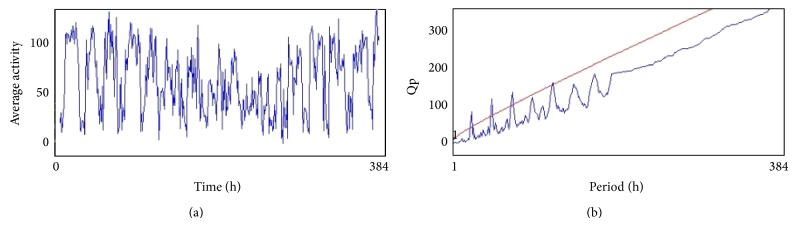
(a) and (b) show the average activity pattern and chi-square periodogram, respectively, for the PD flies exposed to 10^−3^ molar dopamine in diet (*N* = 20).

**Figure 8:**
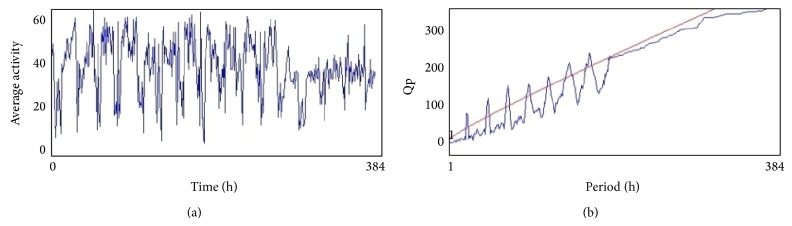
(a) and (b) show the average activity pattern and chi-square periodogram, respectively, for the PD flies exposed to 0.25 *μ*L/mL of* Centella asiatica* leaf extract in diet (*N* = 20).

**Figure 9:**
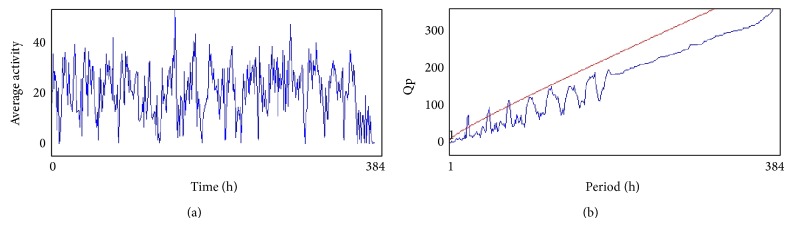
(a) and (b) show the average activity pattern and chi-square periodogram, respectively, for the PD flies exposed to 0.50 *μ*L/mL of* Centella asiatica* leaf extract in diet (*N* = 20).

**Figure 10:**
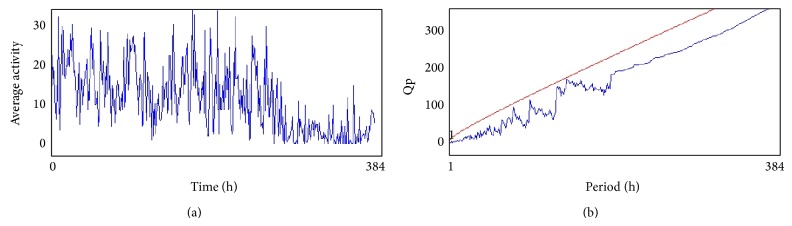
(a) and (b) show the average activity pattern and chi-square periodogram, respectively, for the PD flies exposed to 1.0 *μ*L/mL of* Centella asiatica* leaf extract in diet (*N* = 20).

**Figure 11:**
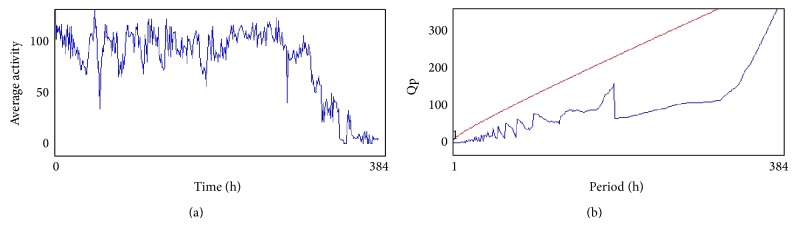
(a) and (b) show the average activity pattern and chi-square periodogram, respectively, for the control flies exposed to 0.25 *μ*L/mL of* Centella asiatica* leaf extract in diet (*N* = 20).

**Figure 12:**
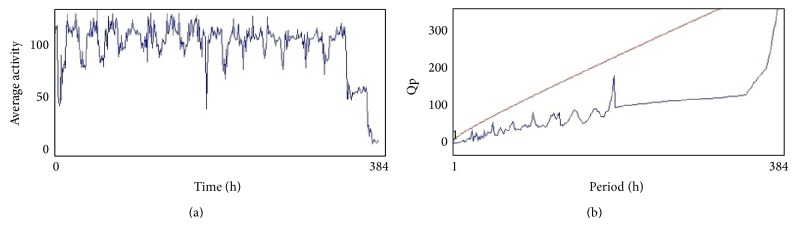
(a) and (b) show the average activity pattern and chi-square periodogram, respectively, for the control flies exposed to 0.50 *μ*L/mL of* Centella asiatica* leaf extract in diet (*N* = 20).

**Figure 13:**
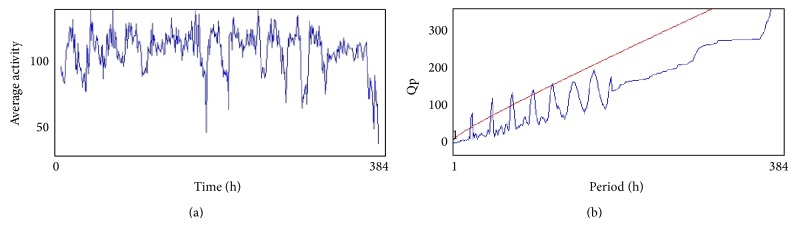
(a) and (b) show the average activity pattern and chi-square periodogram, respectively, for the control flies exposed to 1.0 *μ*L/mL of* Centella asiatica* leaf extract in diet (*N* = 20).

**Figure 14:**
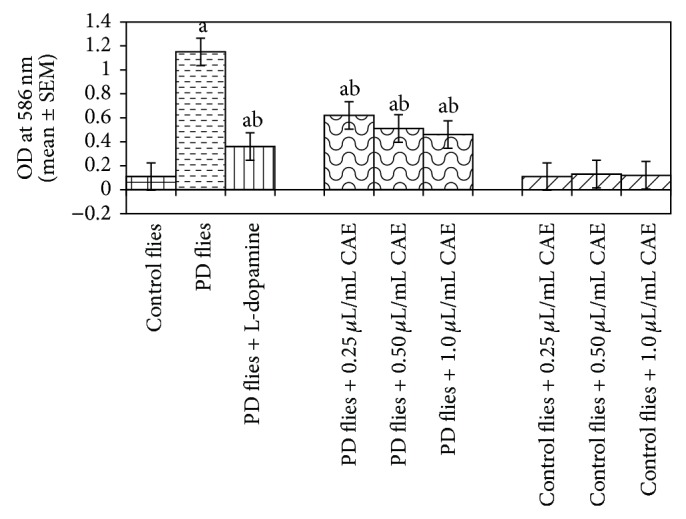
Effect of* Centella asiatica* extract (CAE) on lipid peroxidation measured in the brains of transgenic* Drosophila melanogaster* after 24 days of the exposure in various treated groups (^a^significant with respect to control, *P*-0.01; ^b^significant with respect to PD model flies, *P*-0.05). L-Dopa = 10^−3^ M; control flies: UAS-alpha-syn; PD flies: elav-GAL4; UAS-alpha-syn.

**Figure 15:**
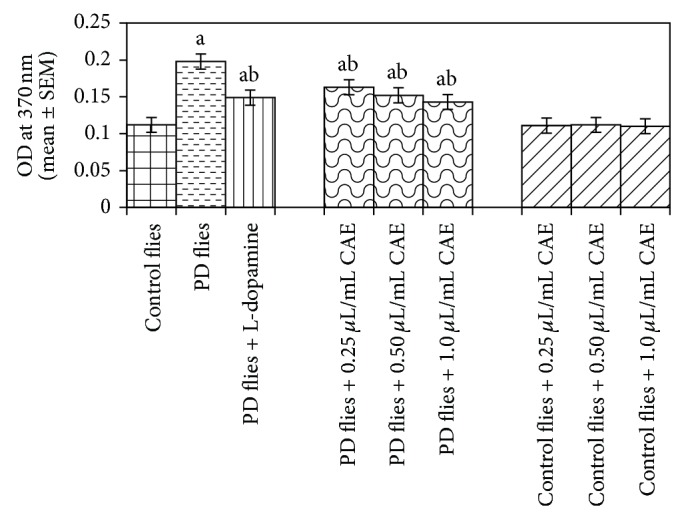
Effect of* Centella asiatica* extract (CAE) on protein carbonyl content measured in the brains of transgenic* Drosophila melanogaster* after 24 days of the exposure to various treated groups (^a^significant with respect to control, *P*-0.01; ^b^significant with respect to PD model flies, *P*-0.05). L-Dopa = 10^−3^ M; control flies: UAS-alpha-syn; PD flies: elav-GAL4; UAS-alpha-syn.

The compounds present in acetone extract of leaves of* C. asiatica* were identified by GC-MS analysis (Figure 1). The GC-MS chromatogram shows the presence of 8 major compounds with highest concentrations. Their retention times (RT), molecular formula, and molecular weight (MW) in the leaves of* C. asiatica* are presented in Table 1. The GC-MS analysis revealed that the acetone extract is mainly composed of *β*-elemene, benzene, 1,1′-oxybis, bicyclotrimethyl-8-methylene, *α*-humulene, *α*-cadinol, 2-hexadecen-1-ol, 3,7,11,15-tetramethyl, and neophytadiene. Figures 2(a)–2(g) show the fragmentation pattern of mass spectrum and structures of compounds extracted from the leaf extract and confirmed through NIST library of GC-MS. Those peaks matching similarity index (SI) greater than 70% in NIST library were assigned. Some of the major peaks are either column bleeding or impurities in plant extract. First the climbing assay was performed for the control and the PD flies. The climbing response of control flies did not change for the 24 days of evaluation but from the day 12, the response of the PD flies was significantly lowered as compare to the control (Figure 3; *P* < 0.05). Hence duration of treatment was selected for 24 days and the climbing assay was performed after 24 days of the exposure to various doses of* C. asiatica* extract. The exposure of PD flies to 0.25, 0.50, and 1.0 *μ*L/mL of* C. asiatica* extract showed a dose-dependent significant delay in the loss of climbing ability (Figure 4; *P* < 0.05). The selected doses of* C. asiatica* did not show any effect on the climbing ability of the control flies (Figure 4). For studying the activity pattern the data collected by* Drosophila* Activity Monitor (DAM) was analyzed by chi-square periodogram. For control flies the number of significant peaks was more (Figures 5(a) and 5(b)) as compared to the PD flies (Figures 6(a) and 6(b)). A dose-dependent delay in the loss of activity pattern was observed in PD flies exposed to 0.25, 0.50, and 1 *μ*L/mL of* C. asiatica* leaf extract (Figures 7(a), 7(b), 8(a), 8(b), 9(a), and 9(b)). No change in the activity pattern of control flies exposed to 0.25, 0.50, and 1 *μ*L/mL of* C. asiatica* extract was observed (Figures 10(a), 10(b), 11(a), 11(b), 12(a), and 12(b)). PD flies exposed to 10^−3^ M of dopamine also showed a delay in the loss of activity (Figures 13(a) and 13(b)). The results obtained for the lipid peroxidation are shown in (Figure 14). A dose-dependent significant decrease in the mean absorbance values for the estimation of lipid peroxidation was observed in the PD flies exposed to 0.25, 0.50, and 1.0 *μ*L/mL of* C. asiatica* extract as compared to untreated PD flies (Figure 14; *P* < 0.05). The exposure of the selected doses of* C. asiatica* extract did not show any increase in the mean absorbance values for the estimation of lipid peroxidation (Figure 14). The results obtained for the protein carbonyl content in the brain of PD flies are shown in (Figure 15). A dose-dependent decrease in the mean absorbance values for the estimation of protein carbonyl content was observed in the flies exposed to 0.25, 0.50, and 1.0 *μ*L/mL of* C. asiatica* extract as compared to untreated PD flies (Figure 15; *P* < 0.05). No increase in the protein carbonyl content was observed in the control flies exposed to selected doses of* C. asiatica* extract (Figure 15).

**Figure 16:**
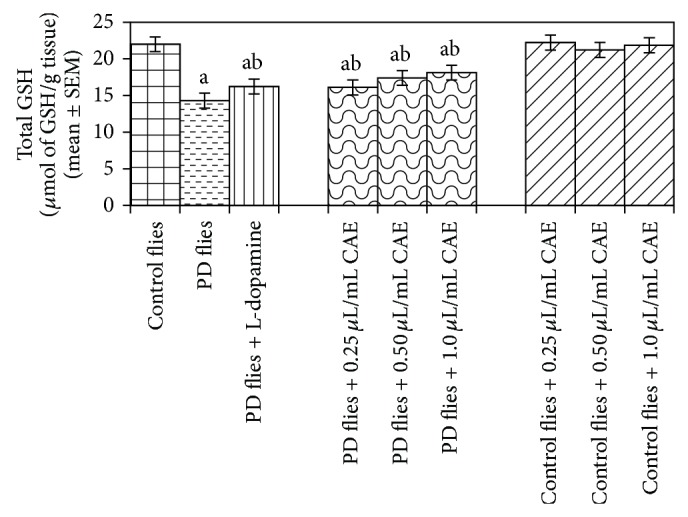
Effect of* Centella asiatica* extract (CAE) on glutathione (GSH) content measured in the brains of transgenic* Drosophila melanogaster* after 24 days of the exposure to various treated groups (^a^significant with respect to control, *P*-0.01; ^b^significant with respect to PD model flies, *P*-0.05). L-Dopa = 10^−3^ M; control flies: UAS-alpha-syn; PD flies: elav-GAL4; UAS-alpha-syn.

**Figure 17:**
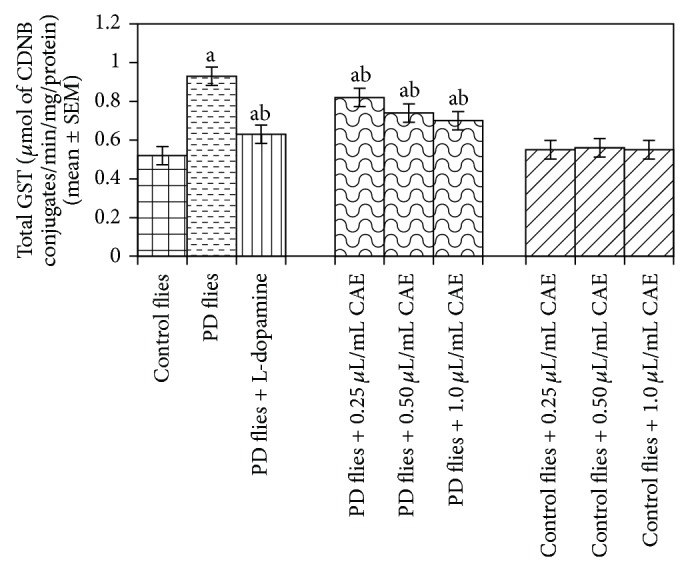
Effect of* Centella asiatica* extract (CAE) on glutathione-S-transferase (GST) content measured in the brains of transgenic* Drosophila melanogaster* after 24 days of the exposure to various treated groups (^a^significant with respect to control, *P*-0.01; ^b^significant with respect to PD model flies, *P*-0.05). L-Dopa = 10^−3^ M; control flies: UAS-alpha-syn; PD flies: elav-GAL4; UAS-alpha-syn.

The results obtained for total glutathione (GSH) content are shown in (Figure 16). A dose-dependent increase in the GSH content was observed in the PD flies exposed to 0.25, 0.50, and 1 *μ*L/mL of* C. asiatica *extract (Figure 16). The control flies exposed to 0.25, 0.50, and 1 *μ*L/mL of* C. asiatica* extract also showed a dose-dependent increase in the GSH content as compared to untreated control flies (Figure 16). The results obtained for the estimation of glutathione-S-transferase (GST) activity are shown in (Figure 17). A significant increase in the activity of GST was observed in PD flies (Figure 17). The exposure of PD flies to 0.25, 0.50, and 1 *μ*L/mL of* C. asiatica* extract showed a dose-dependent decrease in the GST activity (Figure 17). No increase in the GST activity was observed in control flies exposed to 0.25, 0.50,and 1 *μ*L/mL of* C. asiatica* extract (Figure 17).

## 4. Discussion

The results of the present study suggest that the leaf extract of* C. asiatica* is potent in reducing the PD symptoms expressed in the transgenic flies.* C. asiatica* extract at 100, 200, and 300 mg/kg showed a dose-dependent protective effect against the oxidative stress [17]. Asiaticoside, a terpenoid saponin of* C. asiatica,* was found to protect dopaminergic neuron by antagonizing 1-methyl-4-phenyl 1,2,3,6-tetrahydropyridine (MPTP). The MDA content was significantly reduced and the glutathione level was significantly increased after the treatment of asiaticoside [4]. In one study the* C. asiatica* powder was found to reduce the level of low density lipoprotein compared to control rats [2]. Asiatic acid has also been reported to be protective against rotenone or H_2_O_2_ induced cell injury in SH-SY5Y and have been suggested to be developed as an agent for PD therapy [7].

The abundant presynaptic terminal associated *α*-synuclein aggregates are responsible for synaptic pathology and neurodegeneration [18]. The damaging neurons (in PD patients) have been reported to generate hydrogen peroxide and free radicals [19]. In our present study with the model of transgenic* Drosophila* expressing human *α*-synuclein, a significant reduction in the lipid peroxidation and protein carbonyl content was observed in the flies exposed to various doses of* C. asiatica.* The results obtained in our study supports the results obtained by Haleagrahara and Ponnusamy [8] in which the supplementation of* C. asiatica* extract reduced the lipid peroxidation and protein carbonyl content in corpus striatum and hippocampus of rats. The therapeutic intervention that could effectively decelerate the rate of degeneration within the substantia nigra pars compacta could add years of mobility and reduce morbidity associated with PD [19]. Hence the attention has been directed towards the identification of the inhibitors of alpha synuclein aggregations or free radicals scavengers [20].* C. asiatica* extract has been reported to reduce the lipid peroxidation and protein carbonyl content in the brains of aged rats [21]. One of the compounds isolated from* C. asiatica*, that is, madecassoside, was effective in recovering MPTP-induced PD symptoms by its neuroprotective effects including reversing the depletion of antioxidant activity, increasing ratio of BCl-2/Bax, and increasing protein expression of BDNF [22]. Our earlier study with* Eucalyptus citriodora* and* Salvadora persica* leaf extract showed a protective effect in the PD model flies expressing human *α*-synuclein [23, 24]. The natural antioxidants such as apigenin [25], curcumin [11], ascorbic acid [26], gingerol [27], and grape extract [28] potent in delaying the loss of climbing ability and reduced the oxidative stress in the brains of PD model flies. In our GC-MS analysis of *β*-elemene has been reported to have antiproliferative effect for some cancer cells [29]. Humulene has been reported to produce anti-inflammatory effect and also has inhibitory effect on tumor necrosis factor-*α*-/(TNF-*α*) and interleukin-1 *β* (IL1B) [30]. *α*-Cadinol is an antifungal [31] and hepatoprotective [32]. C_14_H_14_O is arachidic acid, a saturated fatty acid [33], C_20_H_40_O_2_ is phytanic acid, a branched fatty acid, and its metabolites have been reported to bind or activate transcription factors [34].

**Figure 18:**
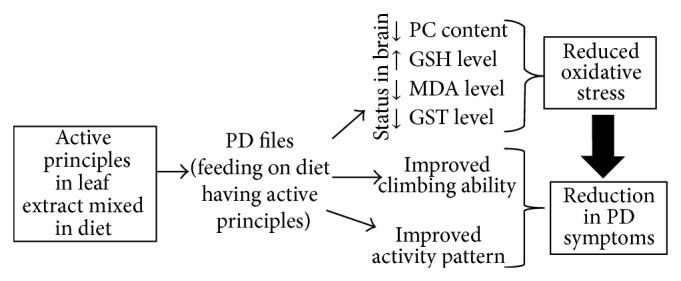
Possible mechanism of protection by* Centella asiatica* leaf extract in Parkinson's disease model transgenic flies.

Various studies on the experimental models suggest that the oxidative stress plays an important role in the neurodegenerative diseases [35]. Hence the lipid peroxidation (LPO) and protein carbonyl (PC) content were measured in the brains of* Drosophila* as a marker of oxidative stress. LPO represents a reliable marker of free radical generation and indicates the membrane damage [36]. Oxidative stress leads to the damage of lipid, protein, and DNA [37]. In our present study, significant changes in the activities of GST and the contents of GSH and MDA confirmed the oxidative stress in PD flies due to synuclein aggregation or Lewy bodies formation. The MDA content indicates the generation of ROS and a significant augmentation in the GST activity observed in PD flies, a response to abate the adverse effect of free radicals may be generated during the progression of disease. The depletion in GSH content was observed in PD flies. This decrease in GSH content is due to the oxidative stress resulting from the aggregation of alpha synuclein. The treatment of control flies with the* C. asiatica* extract results in an increase in the GSH content. The exposure of PD flies to various doses of* C. asiatica* extract results in an increase in the GSH content and decrease in the GST and MDA content. On the basis of results obtained in our present study the possible mechanism for the protection against the PD symptoms by* C. asiatica* extract is shown in (Figure 18). These findings are concurrent with the findings of other workers in which the* C. asiatica* extract has been reported to reduce MDA levels; increase SOD, and GSH levels in various experimental models [38]. The dietary supplementation of the flavonoids showed improvement in cognitive function possibly by protecting vulnerable neurons, enhancing existing neuronal function or by stimulating neuronal regeneration [39]. The complex brain of* Drosophila* is capable of learning and memory. Almost all the major classes of ion channels, receptors, and neurotransmitters similar to humans composed of specialized cell types are found in* Drosophila* brain [40].

## 5. Conclusions

The leaf extract of* C. asiatica* is effective in reducing the oxidative stress in the brains of PD model flies and also delays the loss of climbing ability and activity pattern.

## Figures and Tables

**Table 1:** The active principles in the leaf extract of *Centella asiatica* with their retention time (RT), molecular formula, and molecular weight.

Number	RT	Name of the compound	Molecular formula	MW
1	7.55	*β*-Elemene	C_15_H_24_	204.35
2	7.64	Benzene, 1,1′-oxybis	C_14_H_14_O	198.26
3	7.80	Bicyclo[7.2.0]undec-4-ene, 4,11,11-trimethyl-8-methylene-, [1R-(1R^*x*^,4Z,9S^*x*^)]	C_15_H_24_	204.35
4	8.01	*α*-Humulene	C_15_H_24_	204.35
5	8.19	*α*-Cadinol	C_15_H_26_O	222.37
6	10.20	2-Hexadecen-1-ol, 3,7,11,15-tetramethyl-, [R-[R^*x*^,R^*x*^-(E)]]	C_20_H_40_O	296.531
8	10.36	Neophytadiene	C_20_H_38_	278.515
